# Effect of prenatal cocaine on early postnatal thermoregulation and ultrasonic vocalization production

**DOI:** 10.3389/fpsyg.2013.00882

**Published:** 2013-11-26

**Authors:** Matthew S. McMurray, Philip S. Zeskind, Stephanie M. Meiners, Kristin A. Garber, Hsiao Tien, Josephine M. Johns

**Affiliations:** ^1^Department of Psychology, University of Illinois at ChicagoChicago, IL, USA; ^2^Department of Pediatrics, Levine Children's Hospital at Carolinas Medical CenterCharlotte, NC, USA; ^3^Department of Psychiatry, University of North CarolinaChapel Hill, NC, USA; ^4^Frank Porter Graham Child Development Institute, University of North CarolinaChapel Hill, NC, USA

**Keywords:** prenatal cocaine, thermogenesis, ultrasonic vocalization, cardiac, brown adipose tissue, stress

## Abstract

Prenatal cocaine exposure can alter the postnatal care received by rat pups. Such effects could be caused in part by alterations in pup-produced stimuli that elicit early postnatal maternal care. Pup ultrasonic vocalizations are thought to be a particularly salient stimulus, and when paired with other cues, may elicit maternal attention. Cocaine is known to acutely alter thermoregulatory and cardiac function, thus prenatal cocaine may affect vocalizations through altering these functions. The data presented here determine the impact of full term prenatal cocaine exposure, saline exposure, or no exposure on thermogenic capacity, cardiac function, and the resulting ultrasonic vocalizations across the early postnatal period (days 1–5). Results indicated that while sharing many similar characteristics with saline-exposed and untreated animals, prenatal cocaine exposure was associated with specific alterations in vocalization characteristics on postnatal day 1 (PND 1), including call amplitude. Furthermore, numerous spectral parameters of their vocalizations were found altered on PND 3, including rate, call duration, and frequency, while no alterations were found on PND 5. Additionally, cocaine-exposed pups also showed a reduced thermoregulatory capacity compared to saline animals and reduced cardiac mass compared to untreated animals on PND 5. Together, these findings indicate that prenatal cocaine may be altering the elicitation of maternal care through its impact on vocalizations and thermoregulation, and suggests a potential mechanism for these effects through cocaine's impact on developing stress systems.

## Introduction

Unlike the profound neuroanatomical and behavioral alterations associated with Fetal Alcohol Spectrum Disorder (Kodituwakku, 2009; Norman et al., 2009), those associated with prenatal cocaine exposure are more subtle. These effects are well-documented (Chae and Covington, 2009; Bandstra et al., 2010) and a number may directly impact the ability of the infant to elicit optimal maternal care. Deleterious effects of cocaine exposure on maternal stress responsivity and infant attention have been reported in human clinical populations (Strathearn and Mayes, 2010), as well as in studies of rodent mothers treated with cocaine while pregnant (Kinsley et al., 1994; Vernotica et al., 1996, 1999; Quiñones-Jenab et al., 1997; Johns et al., 1998, 2005; Nelson et al., 1998; McMurray et al., 2008). While there is currently a rich literature demonstrating the effects of cocaine on rodent maternal behaviors, few studies to date have systematically examined how cocaine alters auditory, olfactory, and other stimuli produced by pups that elicit care.

A number of cues produced by infants can modify maternal responses, including ultrasonic vocalizations (USVs) (Smotherman et al., 1974; Brunelli et al., 1994; Farrell and Alberts, 2002a,b; D'Amato et al., 2005; Okabe et al., 2013), odors (Lévy et al., 2004; Okabe et al., 2013), and temperature (Henning and Romano, 1982; Bates et al., 1985; Leon et al., 1985; Adels and Leon, 1986; Woodside and Jans, 1988; Jans and Woodside, 1990; Stern and Lonstein, 1996). The importance of each cue depends to varying extents on the maternal environment, age of the pup producing the cue, and numerous other factors (Champagne et al., 2001, 2003; Mattson et al., 2001; Brudzynski, 2005). Additionally, pup-produced cues likely interact with alterations in maternal perception and response resulting from drug exposure or environmental disruption. As a stimulus for maternal attention, a sustained high-rate of vocalizing by pups is the most effective for eliciting retrieval from dams (Deviterne et al., 1990; Brunelli et al., 1994; Farrell and Alberts, 2002a,b; Zimmerberg et al., 2003; Fu et al., 2007) and can also be an important stimulus for licking (Brouette-Lahlou et al., 1992), an important social behavior. Rats of various ages vocalize in response to handling, cold temperatures, isolation, and social factors (Blumberg et al., 1992; Shair et al., 1997; Branchi et al., 2001; Hahn and Lavooy, 2005); thus, USVs constitute one avenue of communication between pup and mother (Brunelli et al., 1994; Brudzynski, 2005).

Aside from vocalizations, thermoregulation is also an important determinant of maternal attention. Decreases in pup body temperature have been associated with increases in nursing behaviors in rat dams, through which heat is passed to the litter (Henning and Romano, 1982; Bates et al., 1985; Leon et al., 1985; Adels and Leon, 1986; Woodside and Jans, 1988; Jans and Woodside, 1990; Stern and Lonstein, 1996). In the early postnatal period, individual rat pups rely not only on internal metabolic sources for heat production, but also on external sources, such as their littermates and their mother. As a litter, rat pups achieve warmth through huddling, are insulated from cold by the nest, and receive additional heat from their mother during close contact nursing. Isolated rat pups produce heat primarily through brown adipose tissue (BAT) thermogenesis (Smith, 1964; Alberts, 1978), supported by modulation of cardiorespiratory responses (Blumberg et al., 1997). Brown adipose tissue thermogenesis is immediately apparent at birth in rats (Blumberg et al., 1997; Sokoloff et al., 1998), and its disruption may play a role in the development of obesity and diabetes (Cinti, 2006, 2005; Cypess et al., 2009).

Thermal state also has tremendous influence over vocalizing behavior in early life. Aside from emotional and social factors, it has been hypothesized that many USVs produced in the early postnatal period are by-products of mechanisms that sustain cardiorespiratory function (Blumberg and Alberts, 1990; Blumberg and Sokoloff, 2001). Interestingly, such vocalizations do not depend on cortical control (Middlemis-Brown et al., 2005), suggesting that they may be independent from stress-induced vocalizations. It is unknown if characteristics (other than rate) of pup USVs are altered by increasing thermal challenges, although such relationships likely exist given the association between body weight and USV frequency (Blumberg et al., 2000). Regardless, these developmental changes in thermoregulation and vocalizations provide interesting targets of study for investigations of developmental disorders.

The effects of prenatal cocaine exposure on the relationship between pup thermal control, vocalization production, and cardiac function has not been thoroughly investigated. Prenatal cocaine likely alters thermally induced vocalizations through its impact on central serotonin (Ray et al., 2011) and norepinephrine (Madden et al., 2013), metabolism and cardiac rate (Blumberg et al., 1997; Sokoloff et al., 1998), and the developing stress response systems (Yee et al., 2011); all of which independently alter thermoregulation and vocalizations. Additionally, maternal cocaine use has been strongly associated with malnourishment of the mother and fetus, as well as placental vasoconstriction, which further complicates nourishment delivery to the fetus, potentially altering long-term adipose tissue volume and function (Mostyn and Symonds, 2009).

This study aimed to determine if there are differences in thermoregulation, cardiac function, and ultrasonic vocalization production resulting from prenatal cocaine exposure. Additionally, in normal animals decreased body temperature results in increased cardiac rate and vocalization production. Thus, an additional aim of this study was to determine if this normal relationship between these variables is intact following prenatal cocaine exposure. While these effects may have implications on maternal care, that endpoint is not studied here. Given cocaine's effects on thermoregulation in adults, we anticipated disregulation of thermoregulatory processes (including cardiac rate) across the postpartum period, resulting in alterations to the rate of vocalization and potentially more detailed acoustic characteristics (e.g., frequency or amplitude).

## Methods

### Breeding

This study was carried out in strict accordance with the recommendations in the Guide for the Care and Use of Laboratory Animals of the National Institutes of Health. The protocol was approved by the Institutional Animal Care and Use Committee at the University of North Carolina. All efforts were made to minimize suffering throughout the experiment.

Individually housed Sprague-Dawley nulliparous female rats (200 grams, Charles River, Raleigh, NC) were kept on a 12:12 reverse light cycle (8:00 AM dark) for 1 week and then mated until conception was noted by the presence of a vaginal plug and sperm in a vaginal smear (gestation day (GD) 0). Following conception, females were randomly assigned to cocaine, saline, or untreated groups as they became pregnant (see below for treatment information). Weight gain was measured daily for all animals throughout gestation. Water and chow was available *ad libitum* for all except saline-treated rat dams, who were matched with a cocaine dam on a pair-feeding schedule to control for any effects of cocaine-induced anorexia. Seven days following conception (GD 7), females were moved to a colony room and individually housed on a regular 12:12 light:dark cycle with lights on at 7:00 AM. This procedure results in the majority of dams delivering in the normal daylight hours (Mayer and Rosenblatt, 1998). PPD 1 was defined as the calendar day during which delivery was completed. Following delivery, litters were culled to 10 pups (5 males, 5 females) and pups were returned to their own biological mothers.

### Dam treatment

Females were randomly assigned to cocaine, saline, or untreated groups as they became pregnant. Cocaine-treated dams received twice-daily subcutaneous injections of 15 mg/kg of cocaine hydrochloride (calculated as free base, 1 ml/kg/injection total volume, Sigma, St. Louis, MO) dissolved in normal (0.9%) saline at approximately 9:00 AM and 4:00 PM throughout gestation (GD 1–20) and not thereafter. Saline-treated dams received twice-daily subcutaneous injections of 0.9% Saline solution (1 ml/kg/injection). To prevent skin lesions, injections were alternated daily between rear leg flanks. If a lesion appeared, the fur was clipped at the site, cleaned daily with a betadine solution, and a topical antibacterial ointment (Polymycin-Bacitracin-Neomycin, E. Fougera & Co., Melville, NY) was applied to the area. These measures have been shown to minimize skin lesion appearance and severity (McMurray et al., 2008). Untreated control dams received no drug treatment or food restriction during gestation or during the postpartum period, but were weighed daily to control for the effects of handling.

### Apparatus, temperature measurement, and vocalization recording

**Figure 1:**
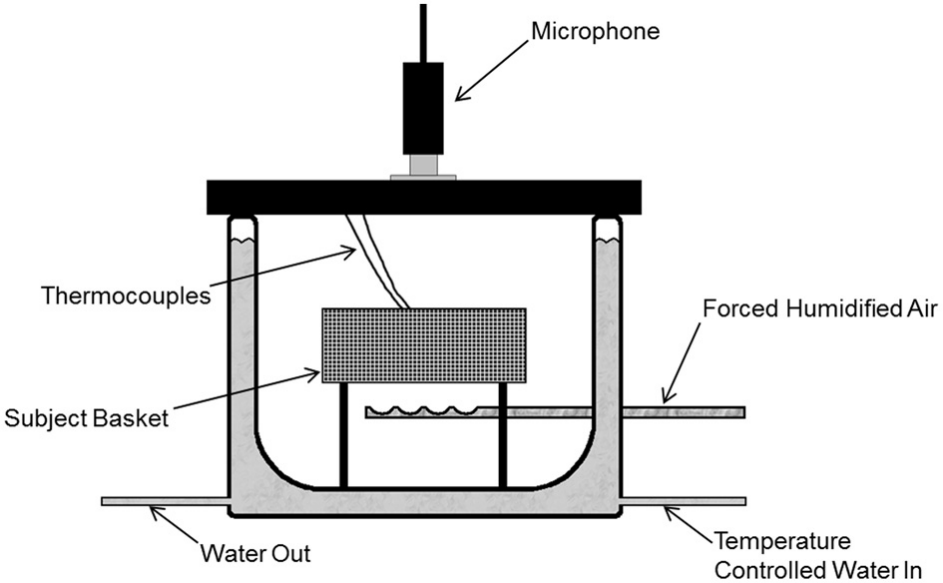
**The thermogenesis testing apparatus.** The subject is placed in the subject basket inside the double-walled glass chamber. The walls of the chamber are filled with temperature-controlled water, which actively regulates the internal temperature of the chamber. Forced humidified air is pumped into the chamber throughout testing. Subject and ambient temperatures were measured using thermocouples.

The apparatus (depicted in Figure 1) consisted of a double-walled glass chamber described previously (Blumberg and Alberts, 1990; Blumberg and Stolba, 1996). Temperature-controlled water was pumped between the walls to control the internal chamber air temperature with a high degree of accuracy. Pups were placed in the chamber on a raised platform constructed of polyethylene mesh, a surface that is only weakly heat conductive and allows for the free passage of air through the chamber. Forced humidified air (300 ml/min) entered the bottom of the chamber, flowed past the pup, and exited through the lid. A mesh wall surrounded the platform to prevent pups from touching the chamber walls directly. Thus, the majority of heat loss by the pup would be convective and less rapid in nature. Holes in the side of the chamber, as well as in its plastic lid, allowed for the connection of thermocouples. Thermocouple leads for measuring physiological and air temperatures were attached to a National Instruments data acquisition device (USB-9211A), which sampled once per second per channel. All hardware and timing was controlled through LabView 2009 software.

Chamber air temperature (*T*_A_) and physiological temperatures were measured using Chromel-Constantan (T-Type) thermocouples (OMEGA Engineering, Inc.; Stamford, CT). *T*_A_ within the metabolic chamber was measured using a thermocouple suspended 2 cm beneath the subject. Two physiological temperatures were acquired by attaching thermocouples just under the surface of the skin using callodion as an adhesive (Spiers and Adair, 1986; Blumberg and Stolba, 1996). One thermocouple was attached in the interscapular region above the brown fat pad, thus providing a measure of interscapular temperature (*T*_IS_) and BAT thermogenesis. The other thermocouple was attached in the lumbar region, and measured the temperature of the back of the subject (*T*_Back_), a non-heat-producing region indicative of general body temperature. The difference between *T*_IS_ and *T*_Back_ (*T*_IS_ - *T*_Back_) was used to confirm the presence and degree of brown adipose tissue thermogenesis (Hull and Segall, 1965; Blumberg and Stolba, 1996).

Ultrasonic recording equipment included model CM16/CMPA40-5V microphones (Avisoft Bioacoustics; Berlin, Germany) connected to a desktop computer through a National Instruments instrumentation recorder (PCI-6132). Microphone voltage was sampled at a rate of 1 MS/s (1 million samples per second) at 14 bit, which allowed for high fidelity recording at frequencies well beyond 100 kHz, more than double the expected fundamental frequency range of 40–50 kHz. Microphones were calibrated prior to each use with the Calibration Unit for Recording Transducers (McMurray and Hubbard, 2013). National Instruments software (LabView, 2009) began acquisition of ultrasonic vocalizations at the session start and terminated at the session end as described below. Recordings were conducted within the thermoregulatory test chambers described above.

### Testing procedure

On PNDs 1, 3, and 5, one pup of each sex with a visible milk band was removed from the litter, weighed, and placed onto a 36°C heat pad for transit to either the testing apparatus (PND 1) or surgical space (PND 3 and 5). To ensure the same pups were not retested at a later developmental time point, they were marked with paw tattoos following testing. Since rat pups exhibit age-dependent thermogenic capacities (Blumberg and Stolba, 1996), age-appropriate thermal challenges were used. Due to the fragile nature of pups on PND 1, thermogenesis was not measured on this day. However, considering the relationship between temperature and vocalization production at this age, a thermal challenge is necessary for the elicitation of vocalizations; thus, pups on this day were rapidly chilled by placing them on a 25°C metal plate for 5 min, during which vocalizations were recorded continuously. Following this, the pup was returned to the incubator and transported back to its litter.

**Figure 2:**
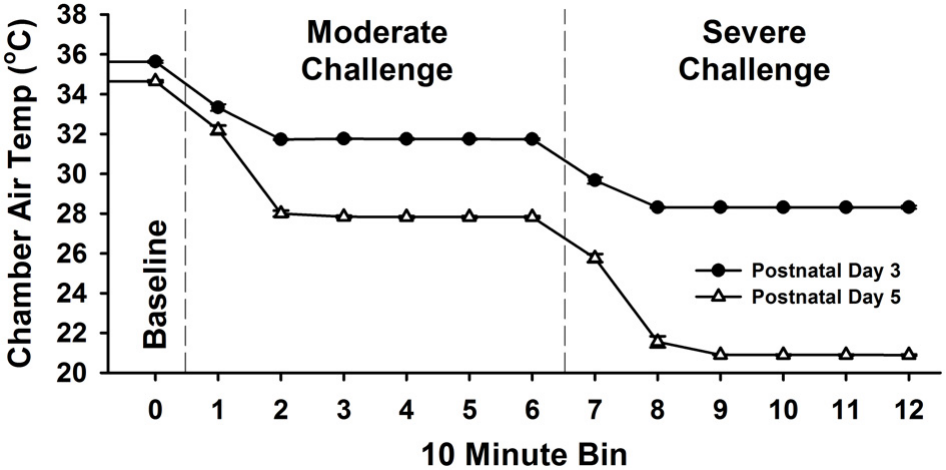
**Environmental challenges posed on postnatal days (PND) 3 and 5.** Subjects were first exposed to a baseline thermoneutral habituation period for 1 h (PND 3: 36°C, PND 5: 35°C), then a developmentally appropriate moderate thermal challenge for 1 h (PND 3: 32°C, PND 5: 28°C), and lastly an extreme thermal challenge for 1 h (PND 3: 28.5°C, PND 5: 21°C).

Across PNDs 3 and 5, pups develop greater thermogenic capacity. Thus, the measurement of thermogenesis becomes relevant and is accomplished via implantable thermocouples. Each pup was anesthetized with isoflurane (5% for induction, 2% for maintenance), thermocouples were implanted 1–2 mm under the skin, and pups were promptly returned to the transportation incubator. This procedure was completed in less than 5 min and subjects were maintained at thermoneutral temperatures throughout surgery using heat pads. After surgery, subjects were transported in the incubator to the test room and placed into the thermal chambers. Data collection began following 60 min of habituation to the chamber at thermoneutral temperatures and continued for 2 h using a series of thermal challenges. Variations in the thermogenic capacities of PND 3 and 5 pups (Blumberg and Stolba, 1996) required the use of age-appropriate thermal challenges (detailed in Figure 2). On PND 3, after 60 min of habituation at 36.0°C, the environmental temperature (*T*_E_) within the apparatus was reduced to 32.0°C (a moderate temperature challenge) for another 60 min, and then again reduced to 28.5°C (an extreme temperature challenge) for a final 60 min. On PND 5, pups were treated in the same manner, except that the habituation temperature was held at 35.0°C, the moderate temperature challenge was 28.0°C, and the extreme temperature challenge was 21.0°C. Biometric data were collected and vocalizations recorded continuously during the tests. Following testing, pups were returned to their litters. The final number of pups tested was 14 cocaine-exposed, 15 saline-exposed, and 17 untreated on PND 1; 30 cocaine-exposed, 28 saline-exposed, and 31 untreated on PND 3; and 31 cocaine-exposed, 34 saline-exposed, and 31 untreated on PND 5.

### Cardiac function assessment

In a separate group of PND 5 animals treated identically to those described above (cocaine and untreated animals only, 10 males and 10 females per group), cardiac function was assessed during the same thermal test. This was done in a separate group of animals, and USVs were not recorded during this experiment. No saline animals were included in this experiment, since they did not differ from cocaine in their thermoregulatory or vocalization responses at this age. Electrocardiograph (ECG) probes (Model 2330, 3M; St. Paul, MN) were applied to the pup's thoracic region, on either side of the interscapular region, with a ground probe applied to the rump region. The data were visually displayed in real-time using an oscilloscope, recorded to the computer via National Instruments hardware (USB-6210), and analyzed to determine average heart rate and R-R intervals from the QRS complex (LabView, 2009). After the test, pups were removed from the chamber and cardiac tissue extracted. After un-anaesthetized decapitation, a thoracic incision was made and the heart was removed, keeping intact the atria and ventricles. After collection, cardiac tissue samples were immediately weighed and frozen at -81°C until time of assay. In order to obtain sufficient tissue for β-adrenergic receptor quantification, cardiac tissue was collected from all subjects, as well as for their eight untested littermates.

#### β-adrenergic receptor quantification

Dissected cardiac tissue was stored at −80^°^C until assayed for β-adrenergic receptor (βAR) levels using a radiolabeled binding assay, as described previously (Mason et al., 1987, 1993). Tissue from four males or four females per group was pooled to obtain sufficient tissue for measurement. A membrane fraction was prepared from heart tissue by homogenization in 6 vol 0.32 M sucrose in 40 mM Tris-HCl buffer (pH 7.4) containing 10 mM MgCl2 and then centrifuged at 900 g for 10 min. The supernatant was spun at 48000g for 10 min. The pellet was washed twice in 40 mM Tris/MgCl2 buffer by rehomogenization and recentrifugation to produce the final P2 fraction pellet. For the 3H-DHA binding assay (relatively equal binding affinities for β1 and β2 receptor subtypes), an aliquot of the tissue was prepared and resuspended in 40 mM TRIS/MgCl2 buffer at a concentration of about 166 mg of the original tissue/ml. These assay incubations were done in triplicate in 1 ml medium containing 40 mM TRIS-HCl/10 mM MgCl2 pH 7.4 and 2 nM 3H-DHA (41.2 Ci/mmol). After the 25 min incubation period at 25°C, samples were filtered on glass fiber discs (0.45 micron pore size, Gelman Inc., Ann Arbor, MI) and washed twice quickly with 5 ml TRIS/MgCl2 buffer. These discs were dried and counted in 5 ml ScintiSafe scintillation mixture with a Beckman LS 7000 scintillation counter at an efficiency of 63%. Non-specific binding was measured using a competing unlabeled ligand (10–6 M propranolol). Due to the very limited amount of heart tissue per animal, pooled tissue was assayed at a single 3H DHA concentration of 2 nM. Since no significant differences were seen at this concentration near the Kd value, scatchard type analysis was not performed. Single point data were used to compare mean receptor binding between treatment groups.

### Data analysis

Although males and females typically had different body weights, after including weight in our statistical models, no consistent sex differences were apparent in temperature or vocalization production. Therefore, males and females were combined into a single group.

Linear mixed models accounting for the clustering of pups coming from the same dam were used to compare the repeated measures of gestational data and subject body weight. *T*_IS_, *T*_Back_, and *T*_IS_ − T_Back_ data were binned into 10-min intervals for statistical and presentation purposes, and for comparisons between treatment groups and sexes. No statistical comparisons between PND 3 and 5 could be made, because of the different age-appropriate thermal challenges used for each time point. To reduce the total number of statistical comparisons, the focus of our analysis was on temperatures from the two thermal challenges. Thus, data from the first 50 min of unchallenged thermal habituation were excluded from our statistical models. Data from the last 10-min bin of the habituation period was included in figures as an estimate of baseline temperature. Remaining data are presented as change from baseline temperature. Means and standard deviations are presented in figures, and while all data are discussed in text, only treatment-specific data are presented in figures.

The ultrasonic vocalization measures analyzed included likelihood to call, number of vocalizations produced, and duration of each call. Additionally, acoustic spectral properties were examined, including measures of pitch (highest and lowest frequency achieved by the fundamental frequency), acoustic power (maximum amplitude), number of harmonics visible (additional waveforms visible at multiples of the fundamental frequency), and the variation in amplitude and frequency of each call (total range of amplitude or frequency achieved by the call). Subjects produced a low average number of vocalizations on all three time points. Generalized estimating equations accounting for the clustering of pups coming from the same dam (litter effects) were used to analyze the ultrasonic vocalization data. Specifically, Chi-squared tests were used to address the likelihood to call while Poisson regression was used to compare the number of vocalizations produced by cocaine, saline, and untreated groups, and linear mixed models were used to evaluate the duration of each call and acoustic spectral differences. All models adjusted for sex, group, body temperature (back or interscapular), and body weight within each PND (1, 3, or 5).

Similar to the thermal data, ECG data (rate and R-R Intervals) were condensed to 10-minute bins, and examined using repeated measures general linear models. Additionally, these data were correlated with thermal data using Pearson correlations, and compared using Fisher's r-z transformation. Cardiac weight and βAR levels were compared between groups using two-tailed *t*-tests.

## Results

### Gestational effects

Gestational data are detailed in Table 1. There were statistically significant differences in gestational weight gain of the dam [*F*_(2, 54)_ = 7.57, *p* = 0.001], with cocaine-treated dams gaining less weight over gestation than untreated (*p* = 0.001) and saline-treated dams (*p* = 0.048); however, cocaine-treated and saline dams gained more weight over the early postpartum period (PPDs 1–5) [*F*_(2, 46)_ = 10.276, *p* = 0.001] than untreated dams (cocaine: *p* = 0.001; saline: *p* = 0.004). There were no statistically significant differences in gestational length, total number of pups in the litter, male to female pup ratio, or pup weights on PNDs 1, 3, or 5.

**Table 1:** **Gestational measures and litter characteristics following maternal cocaine or saline exposure and in untreated animals**.

**Treatment**	**No. of**	**Gestational**	**Postpartum**	**No. of**	**No. of**	**No. of**	**Culled litter**
**group**	**dams**	**weight gain (g)**	**weight gain (g)**	**pups**	**male pups**	**female pups**	**weight (g)**
Cocaine	17	123.5 ± 14.5^*^	15.5 ± 11.3^†^	12.9 ± 1.9	6.5 ± 2.1	6.5 ± 2.1	60.3 ± 6.8
Saline	17	139.9 ± 24.0	14.5 ± 8.7^†^	14.0 ± 2.2	6.9 ± 1.6	7.1 ± 2.2	65.7 ± 4.1
Untreated	24	148.0 ± 19.7	2.1 ± 8.6	13.8 ± 2.2	6.7 ± 1.8	7.1 ± 2.4	67.7 ± 5.5

* Cocaine group differed from Saline (p = 0.048) and Untreated (p < 0.001).

† Cocaine (p < 0.001) and Saline (p = 0.004) differed from Untreated.

### Postnatal day 1

**Figure 3:**
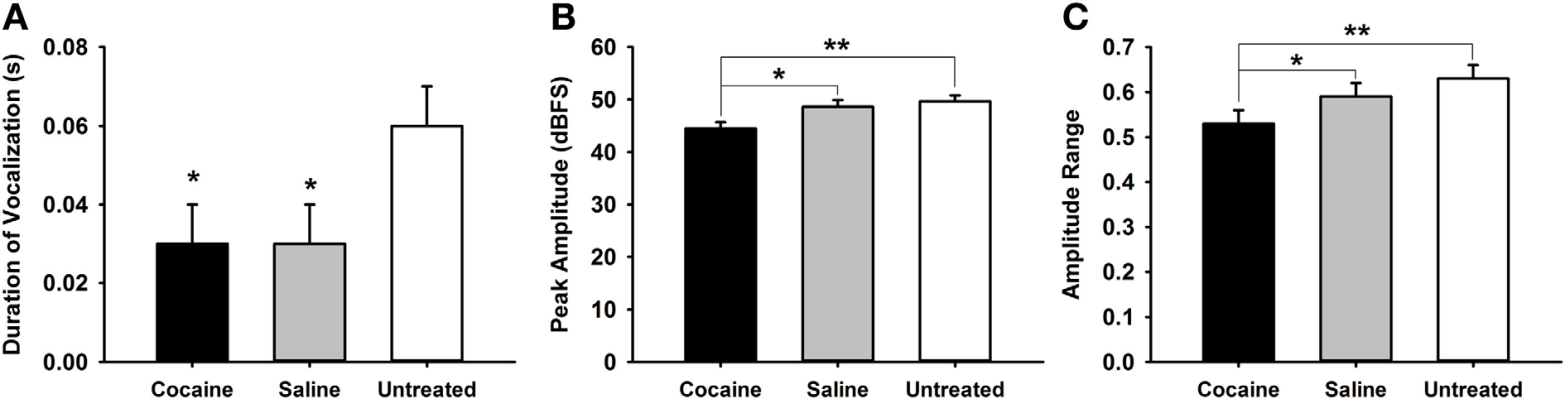
**Postnatal day 1 ultrasonic vocalizations during a 5 min thermal challenge.** Subjects were rapidly chilled by placing them on a 25°C metal plate for 5 min, during which vocalizations were recorded continuously. Pups exposed to prenatal cocaine or saline produced calls with shorter durations than untreated pups **(A)**. Prenatal cocaine exposure also resulted in lower maximum amplitudes **(B)** and a smaller range of amplitudes **(C)** compared to both untreated and saline groups. (^*^p ≤ 0.05. ^**^p ≤ 0.01).

On postnatal day 1, pups underwent a short but severe thermal stressor (25°C), during which USVs were recorded and following which the skin temperature of the pup's rump was measured. Vocalization data are presented in Figure 3. Skin temperature data are not presented, because rump temperature did not differ between treatment groups at the conclusion of testing. Not all pups vocalized on PND 1 (not significant between groups); however, among those that did vocalize, the number of calls produced during the 5 min challenge did not differ between groups. Additionally, pup sex and body weight were not associated with any differences in vocalization parameters. The USVs produced by cocaine pups showed a number of qualitative differences from those produced by untreated and/or saline pups. As shown in Figure 3, untreated pups produced calls with longer durations than both cocaine- (*p* ≤ 0.05) and saline-exposed pups (*p* ≤ 0.05). Additionally, cocaine exposed pups produced calls with lower average peak amplitudes compared to untreated (*p* ≤ 0.01) and saline-exposed pups (*p* ≤ 0.05), and a reduced amplitude range per call compared to untreated (*p* ≤ 0.01) and saline-exposed pups (*p* ≤ 0.05).

### Postnatal day 3

On postnatal day 3, pups underwent a longer duration test than on postnatal day 1, in which thermogenesis (as indicated by *T*_IS_−T_Back_) and concurrent USVs were assessed over a 3 h period across decreasing environmental temperatures. In this environment, pup body weight had a significant impact on both thermogenesis and USV production. Larger pups had significantly higher *T*_IS_ (*p* = 0.05) over the course of the experiment, but not *T*_Back_, reaffirming that heavier pups have greater thermogenic capacity. Pups that weighed more were also more likely to produce one or more USVs during the experiment (*p* = 0.01), and the calls produced had lower peak frequency of the fundamental (*p* = 0.01), lower minimum frequency of the fundamental (*p* = 0.01), a greater number of harmonics per call (*p* = 0.01), higher amplitude (*p* ≤ 0.01), and larger standard deviations of both frequency and amplitude (*p* ≤ 0.01). Despite the impact of weight on these parameters, and the fact that males weighed statistically more than females (*p* ≤ 0.01) on this day, males did not differ statistically from females on any USV measure.

Aside from body weight, the *T*_Back_ of an individual pup was also strongly associated with the vocalizations produced by a pup. Pups with lower *T*_Back_ during the experiment produced more calls (*p* ≤ 0.01), calls with longer durations (*p* ≤ 0.01), and their calls had a larger standard deviation of frequency (*p* ≤ 0.01).

**Figure 4:**
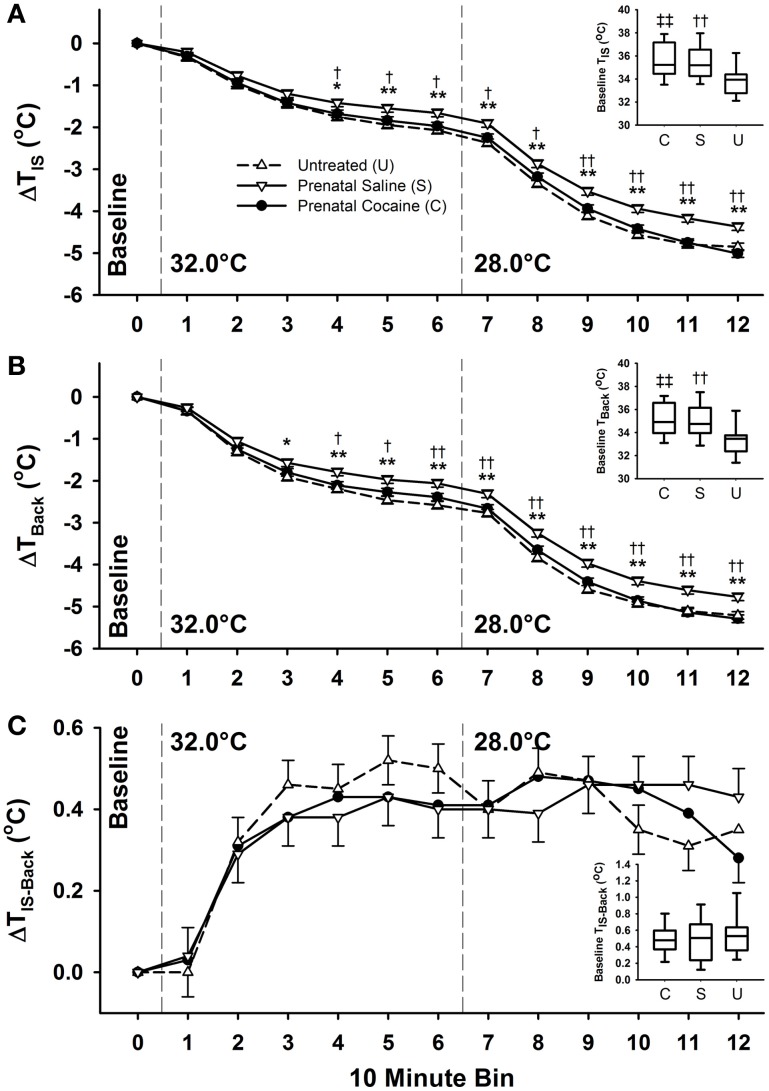
**Postnatal day 3 thermoregulation during a thermoneutral baseline period (36°C), 1 h moderate (32°C), and 1 h extreme (28°C) thermal challenge.** All data are presented as change from baseline. Prenatal saline exposed animals showed *T*_IS_ **(A)** and *T*_Back_ **(B)**, but no change in *T*_IS-Back_ **(C)** compared to both cocaine-exposed and untreated animals. Inset figures show that while cocaine exposed pups did not differ from untreated animals in their change from baseline temperatures, they, along with saline-exposed animals, did statistically differ from untreated animals in their *T*_IS_ and *T*_Back_ baseline temperatures (Saline vs. Cocaine: ^*^p ≤ 0.05, ^**^p ≤ 0.01; Saline vs. Untreated: ^†^*p* ≤ 0.05, ^††^*p* ≤ 0.01; Cocaine vs. Untreated: ^‡‡^*p* ≤ 0.01).

Treatment differences in thermogenesis on postnatal day 3 are presented in Figure 4. Cocaine-exposed pups had generally lower body weights on this day than untreated pups (*p* ≤ 0.05), but did not differ from saline-treated. Given the complex relationship between body weight, thermoregulation, and USV production described above, body weight was included in the statistical model assessing temperature differences between treatment groups. After adjusting for body weight, both cocaine- and saline-exposed pups showed higher baseline *T*_IS_ (*p* ≤ 0.05) and *T*_Back_ (*p* ≤ 0.01) temperatures compared to untreated pups (see Figure 4, insets), but not *T*_IS−Back_. These raw temperature differences were maintained across all thermal challenge periods (all bins *p* ≤ 0.01). Since cocaine- and saline-exposed pups showed chronic increases in temperature across all environmental conditions, change from baseline temperature was also examined to determine if pups reacted to environmental temperatures differently despite this increase. Indeed, saline-exposed pups tended to show a reduced loss of body temperature (*T*_IS_ and *T*_Back_), as would be expected by their increased body temperatures; however, cocaine-exposed pups did not maintain their body temperature to a similar extent, and instead were comparable to untreated pups in the amount of heat lost during the thermal challenges. Importantly, the difference in *T*_IS_ and *T*_Back_ temperatures between the groups was not due to a change in thermogenic capacity (*T*_IS−Back_). Such results may indicate that all groups were in a maximal heat producing state, and implies that variations in heat loss between groups may instead be responsible for the differences we see here.

**Figure 5:**
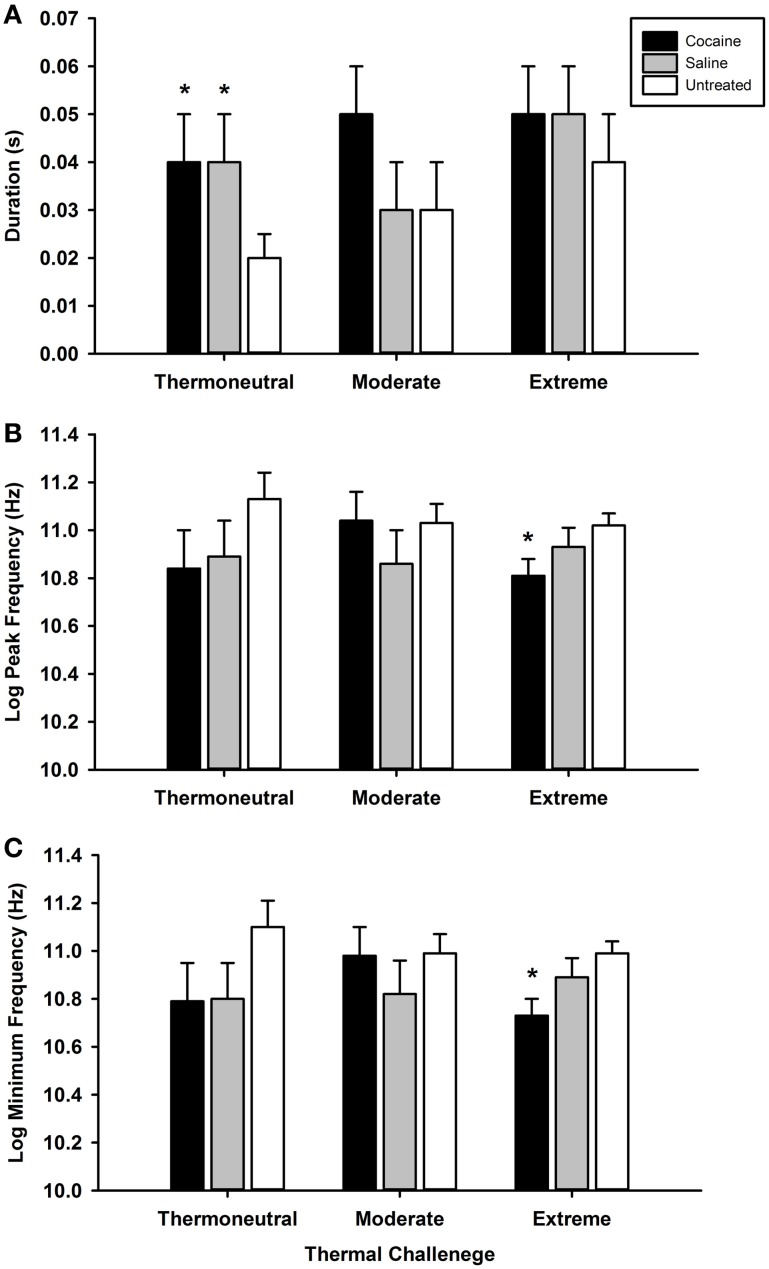
**Postnatal day 3 ultrasonic vocalization characteristics.** Vocalizations were recorded during thermoneutral (35°C), moderate (32°C), and extreme (28°C) thermal challenge periods (data averaged across each period). At this age, pups exposed to prenatal cocaine or saline demonstrated an increased average duration of each call during the thermoneutral period **(A)** compared to untreated animals. Additionally, cocaine exposed animals showed a reduced average maximum **(B)** and minimum **(C)** frequency per call during the extreme thermal challenge period compared to untreated animals (^*^p ≤ 0.05).

Along with alterations in pup temperature, cocaine- and saline-exposed pups showed a myriad of effects on USVs, which are displayed in Figure 5. Many pups did not call at all during the baseline or thermal challenge periods. Only 40% of cocaine, 37% of saline, and 80% of untreated animals vocalized [χ^2^_(2)_ = 3.56, *p* = 0.17]. Of those pups that did produce a call, there was no difference in the number of calls produced. However, as shown in Figure 5A, cocaine- and saline-exposed pups produced vocalizations with longer durations than untreated pups (*p* ≤ 0.05) during thermoneutral periods. Additionally, during the extreme thermal challenge cocaine-exposed pups vocalized with lowered peak frequency (*p* ≤ 0.05), lowered minimum frequency (*p* ≤ 0.01), and with a larger average standard deviation of frequency within a call (*p* ≤ 0.01) compared to untreated pups, but not saline-exposed pups.

### Postnatal day 5

On PND 5, pups underwent a very similar experiment to the one used on postnatal day 3, during which thermogenesis and USV production were assessed over a series of thermal challenges. Again, heavier pups exhibited higher *T*_IS_ (*p* ≤ 0.01) and *T*_IS−Back_ (*p* ≤ 0.05), but not *T*_Back_ measurements, again demonstrating the greater thermogenic capacity of heavier pups. PND 5 pups with higher body weights also produced calls with longer durations, lower peak frequency of the fundamental, lower minimum frequency of the fundamental, lower fundamental frequency at loudest portion of the call, and higher peak amplitude of the call. However, the body weight difference between cocaine and untreated pups seen on PND 3 was no longer apparent on PND 5. Additionally, and as seen on PND 3, males on this day weighed more than females (*p* ≤ 0.01); however, this difference did not result in alterations in USV production.

**Figure 6:**
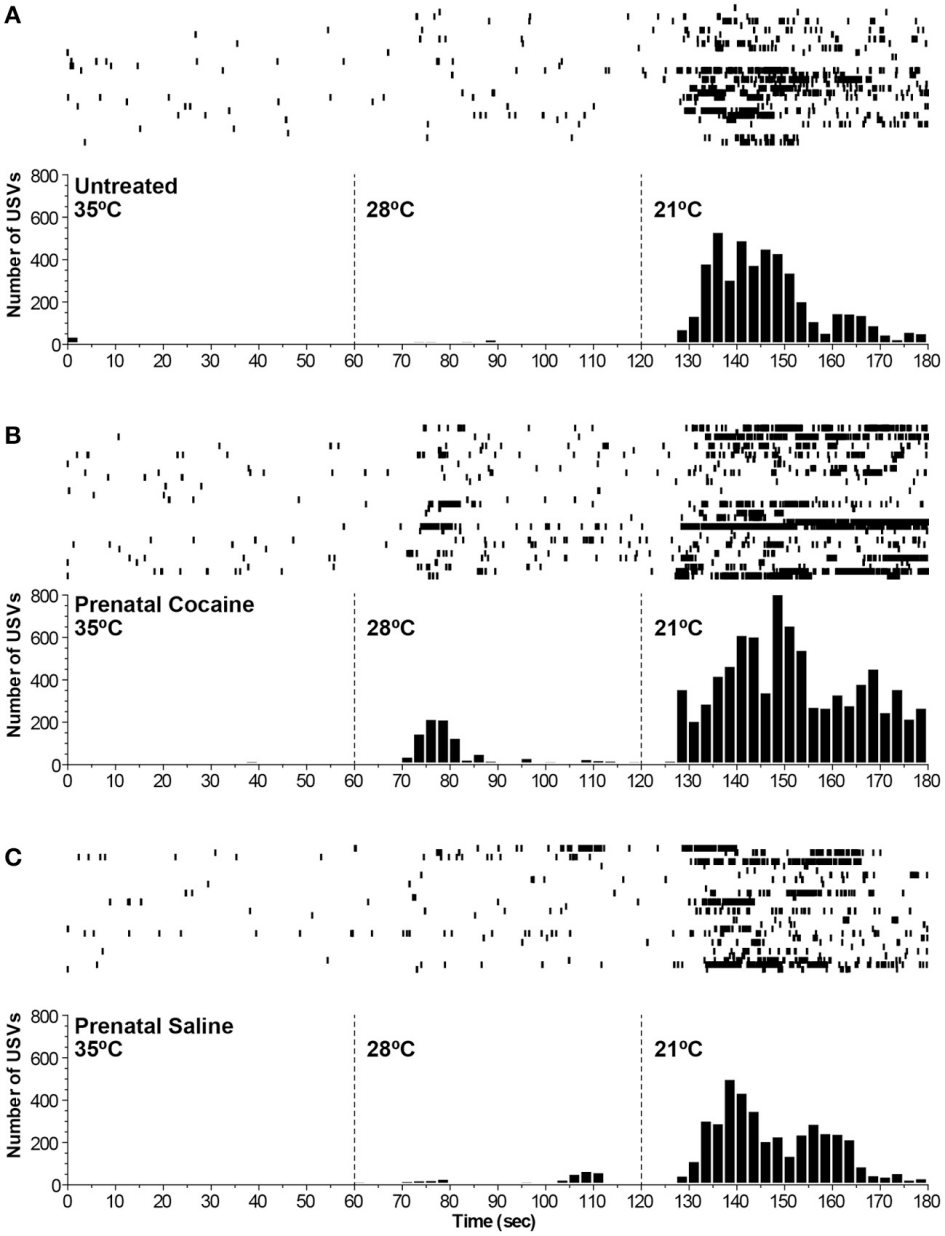
**Raster and histogram depicting the number of ultrasonic vocalizations produced by pups on postnatal day 5 across the 3 h thermal testing period.** The first hour was at baseline thermoneutral temperature (35°C), the second hour was at a moderate thermal challenge (28°C), and the third hour was at an extreme thermal challenge (21°C). Each line of the raster represents a single animal's vocalization pattern, with the histogram below summarizing the data across animals. All groups show relatively similar patterns of vocalizing, with the strongest increase in vocalization rates during the extreme thermal challenge. Although the cocaine **(B)** and saline **(C)** groups both show increases in vocalizing during the moderate thermal challenge period (ns), these increases appear to be driven by only a few subjects and are not seen in untreated animals **(A)**.

During the thermal challenges, the rate of USV production increased as environmental temperature decreased (see Figure 6). During the baseline thermoneutral period, very few calls were seen at all. Pups with higher *T*_Back_ across both thermal challenges produced fewer calls (*p* ≤ 0.01) and the calls produced had shorter durations (*p* ≤ 0.01), lower peak fundamental frequency (*p* ≤ 0.01), lower minimum fundamental frequency (*p* ≤ 0.01), lower frequency at peak amplitude of the fundamental (*p* ≤ 0.01), lower peak amplitude of the fundamental (*p* ≤ 0.01), and a smaller standard deviation of frequency (*p* ≤ 0.01).

**Figure 7:**
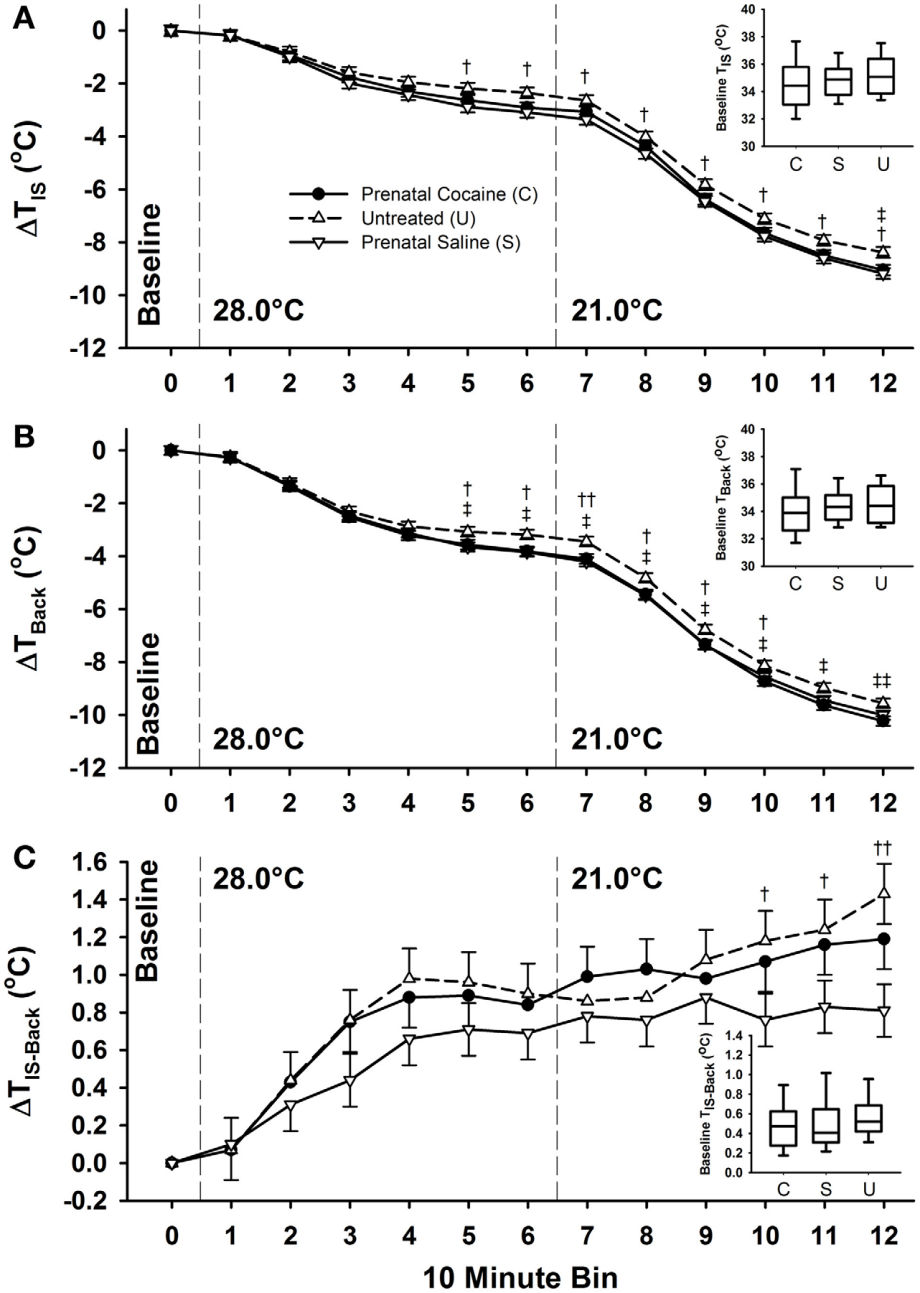
**Postnatal day 5 interscapular and back temperatures during a thermoneutral baseline period (34°C), 1 h moderate (28°C), and 1 h extreme (21°C) thermal challenge.** Prenatal cocaine exposure was associated with an increase in both interscapular **(A)** and back **(B)** temperatures across the later portions of the moderate challenge, and the entirety of the extreme thermal challenge, but no significant difference in *T*_IS-Back_ **(C)**. Additionally, there was no difference between group baseline temperatures (inset graphs). (Saline vs. Untreated: ^†^*p* ≤ 0.05, ^††^*p* ≤ 0.01; Cocaine vs. Untreated: ^‡^*p* ≤ 0.05, ^‡‡^*p* ≤ 0.01).

As was done on PND 3, individual differences in body weight were included in the statistical model examining treatment differences in thermogenic capacity on PND 5. As shown in Figure 7 (insets), there were no baseline *T*_IS_, *T*_Back_, or *T*_IS−Back_ differences due to prenatal cocaine or saline exposure. During the majority of the moderate thermal challenge period (28.0°C) and the entire extreme (21.0°C), both prenatal cocaine and saline exposure resulted in lower *T*_IS_ and *T*_Back_, compared to untreated animals (bins 5–12, *p* ≤ 0.05 or *p* ≤ 0.01, see Figure 7). However, this reduction was not associated with differences in *T*_IS−Back_. Despite the differences in *T*_IS_ and *T*_Back_, there was no difference in the number of calls produced between treatment groups, and no difference in any measure of call characteristics.

### Postnatal day 5 cardiac function

**Figure 8:**
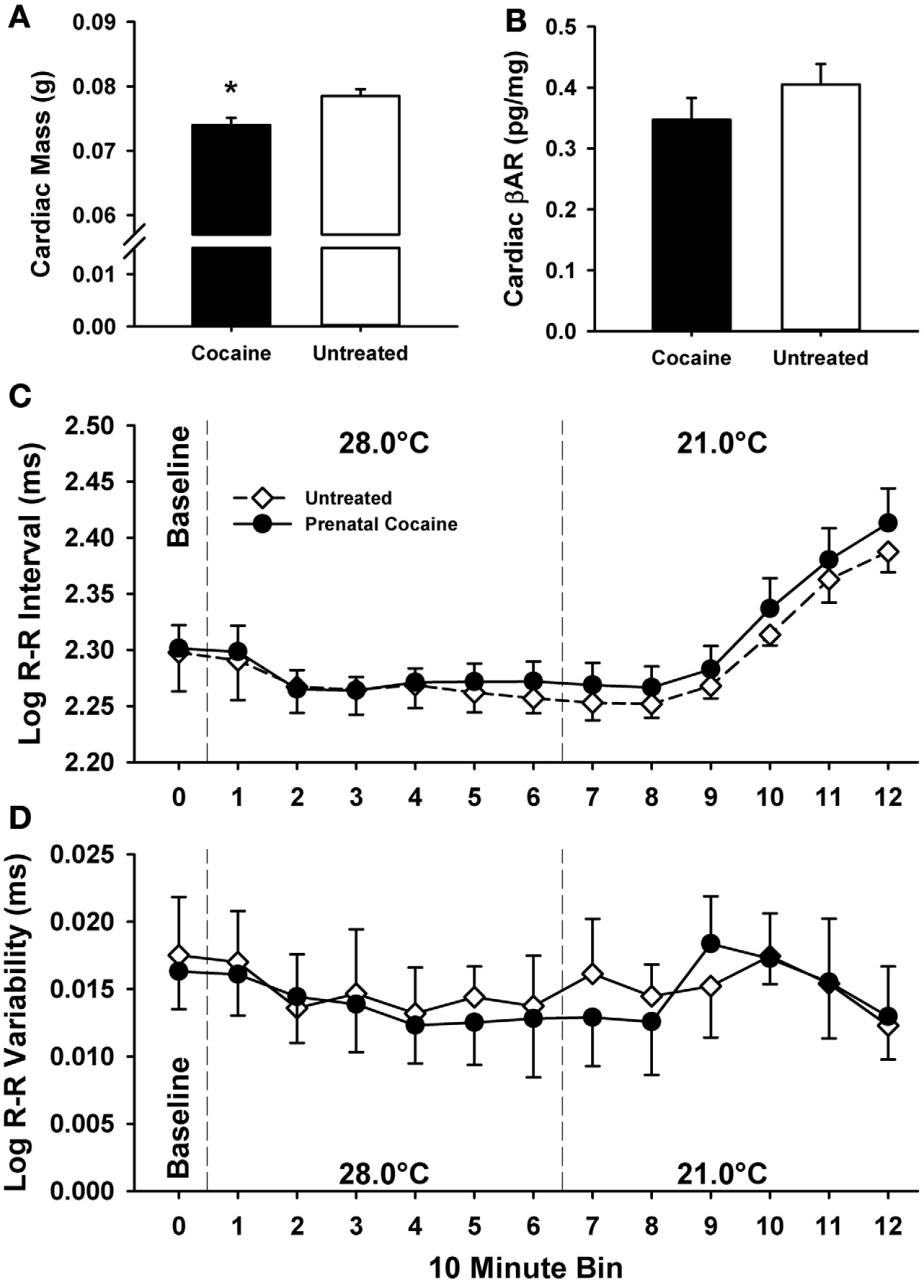
**Assessment of cardiac function on postnatal day 5 during a thermoneutral baseline period (35°C), 1 h moderate (28°C), and 1 h extreme (21°C) thermal challenge.** Prenatal cocaine exposure was associated with decreases in cardiac mass **(A)**, but no change in cardiac β-Adrenergic receptor concentrations **(B)** as measured following the thermal challenges. Additionally, prenatal cocaine exposure was not associated with significant change in heart rate (data not shown), R-R Interval **(C)**, or R-R Interval Variability **(D)** as measured during the thermal challenges. (^*^p ≤ 0.05).

**Figure 9:**
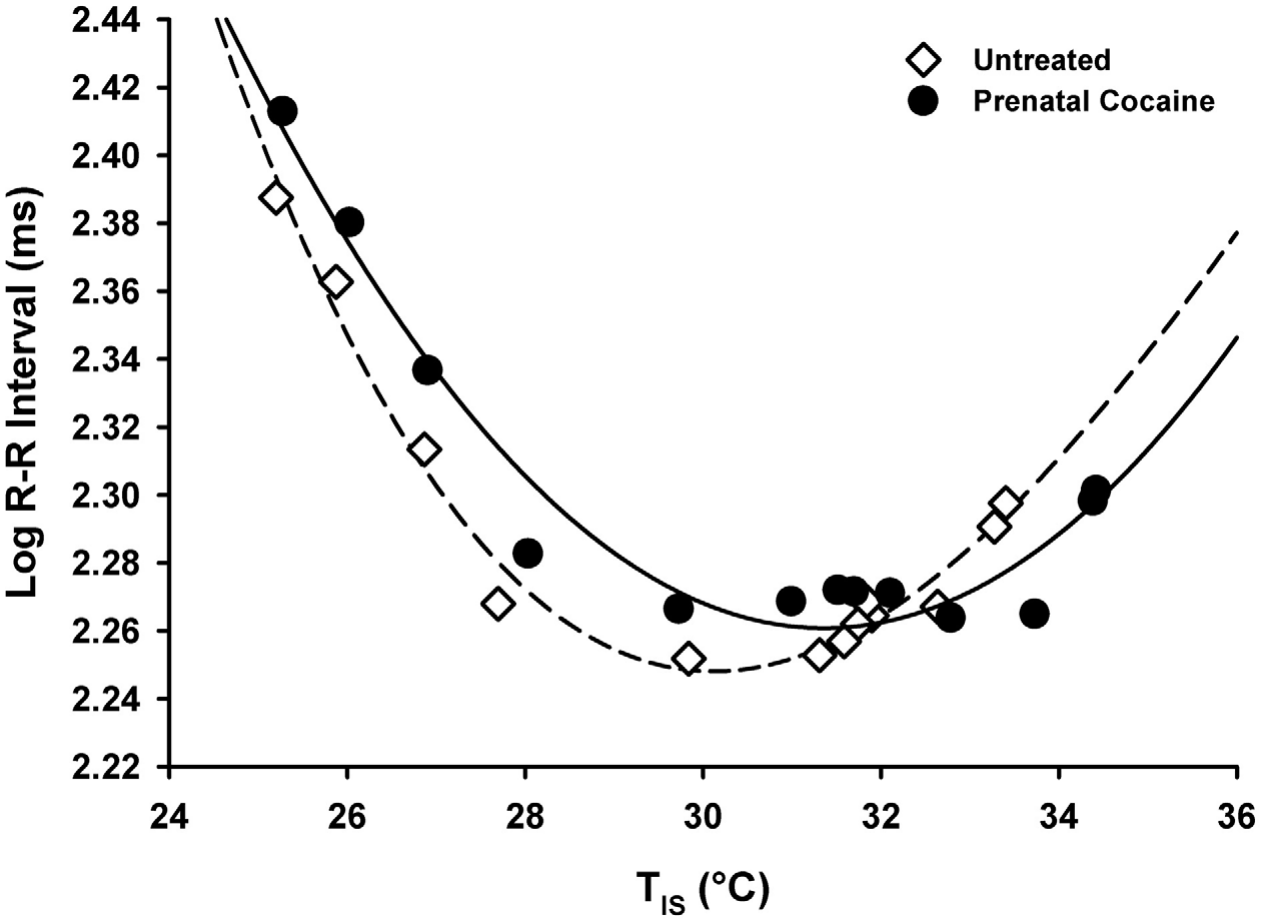
**The relationship between the cardiac R-R Interval and the interscapular temperature of the pup.** Average group temperature and R-R interval for each 10-min bin are presented. An increasing value on the Y-axis indicates a slowing of the cardiac rate. Thus, at colder temperatures, pups tend to have reduced heart rates. Both cocaine-exposed and untreated pups show a similar relationship between these variables, with no significant difference between the two groups.

In a separate group of pups, cardiac function was assessed during and following the thermal challenges used in the above experiment on PND 5. There were no differences between males and females on any cardiac function measure. Treatment differences in cardiac function are visualized in Figure 8. Prenatal cocaine exposure was not associated with alterations in Heart Rate (data not shown), R-R Interval, R-R Variability, or cardiac βAR levels (see Figures 8B,C,D). However, as shown in Figure 8A, cardiac mass was significantly lower in cocaine-exposed pups than in untreated pups [*F*_(1, 127)_ = 8.13, *p* < 0.01]. Additionally, as shown in Figure 9, the relationship between the cardiac R-R Interval and *T*_IS_ was best fit by a 2nd order polynomial function for both cocaine exposed (*r*^2^ = 0.95) and untreated animals (*r*^2^ = 0.98), and did not differ between treatment groups.

## Discussion

The data presented here demonstrate that prenatal exposure to cocaine alters measures of early postpartum thermoregulatory ability and vocalizing behavior, and that it does so similarly in both male and female rat pups, although the pattern of effects differed dynamically depending on the developmental age of the animal and the comparison control groups. There appeared to be effects attributable to perhaps stress (injection and food restriction of dams) as well as separate effects of cocaine exposure itself. These differences are important as they can help direct future mechanistic studies. Thus, here we initially discuss the effects on the saline group, followed by differences in untreated and cocaine exposed offspring. Historically, rodent studies of cocaine exposure have required comparisons to both untreated and saline/food yoked groups to control for anorectic effects of cocaine and injection stress. Results of the effects have been mixed depending on what behaviors are studied. Our inclusion of the saline control was meant to account for many of these factors; however, the pattern of effects seen in our saline-exposed offspring surprisingly differed from both our untreated control and cocaine-exposed animals, especially on PND 3. On this day, saline exposed pups demonstrated baseline body temperatures almost identical to cocaine-exposed animals, but showed greater resilience in response to changes in environmental temperature (reduced change from baseline temperature). Additionally, despite differing from cocaine animals in their temperature regulation, they showed a very similar pattern of effects on vocalizing behavior, with increased call duration and mildly reduced frequency (ns).

Prenatal stress alone has been shown to have numerous effects on cardiovascular development, thermoregulation, and stress responsivity (Hashimoto et al., 2001; Mastorci et al., 2009), but the degree of overlap with cocaine's effect on these measures is unknown. The obvious stress from injections may not be the sole driving factor behind these results, and is likely compounded by the stress of forced food restriction. Although not quantified in the current study, during data collection, the food restricted animals tended not to eat all the food offered, and would instead hide some throughout the cage, exhibiting a hoarding-like behavior. This unusual behavior is likely indicative of a highly stressed state. Thus, the total effect of the compounding of both stressors likely differs from the effects of cocaine, which only featured the stress of injections (reduced appetite likely differs from forced food restriction). Other studies from our own lab and others have shown similar confusing results using this and similar control groups (Wilkins et al., 1998; Johns et al., 2005; Malanga et al., 2007; McMurray et al., 2008), calling into question its usefulness in modeling these specific stress confounds.

Cocaine-exposed offspring, as compared to unexposed offspring, vocalized with shorter call durations and reduced call amplitudes on PND 1, but only 2 days later (PND 3) exhibited increased call durations and reduced frequencies, with no change in amplitude. These early differences in vocalizing behavior were not apparent by PND 5, although vocalization effects have been shown at later developmental ages (Cox et al., 2012). Clearly these important early time points in development are highly dynamic, involving the pup's own developmental trajectories as well as social feedback from mothers and littermates. Indeed, cocaine-exposed pups have been shown to receive less direct contact from dams in these very early time points, regardless of dam drug exposure (Johns et al., 2005). However, the social significance of variations in rodent USVs has yet to be fully elucidated, thus the full importance of our findings is unclear at this time.

Cocaine-exposed pups also showed differences in thermoregulatory ability. On PND 3, the vocalization differences described above were associated with an increase in baseline temperature compared to untreated controls, but no difference in change from baseline temperatures compared to untreated controls. Like the vocalization results, baseline temperature differences seemed to have resolved only two days later on PND 5; however, on that day cocaine-exposed animals did show greater change from baseline temperatures (except *T*_IS−Back_). This was highly unexpected, as the typical relationship between body temperature and vocalizing behavior would suggest that reductions in body temperature should be associated with increased vocalizing (Blumberg et al., 1992). Under this model, the reductions in body temperature seen in cocaine-exposed animals should have been associated with increased vocalizing behavior, but this was not the case.

Body temperature regulation is a relatively unstudied effect of prenatal cocaine, but the presence of such effects is not necessarily surprising given the disturbances in cardiac development previously reported (Regalado et al., 1996; Sun et al., 2003) and prior reports of acute cocaine altering thermoregulation in adults (Crandall et al., 2002). On PND 3, the baseline *T*_IS_ and *T*_Back_ of cocaine-exposed pups was almost 2° (Celsius) warmer than untreated pups on average, and was maintained despite alterations in environmental temperatures. However, this result must be interpreted with caution. Both treatment groups were handled in the same manner, and given 1 h to acclimate to thermoneutral temperature (36°C); however, the untreated pups failed to reach the same baseline temperature as the cocaine-exposed animals. It is unclear if cocaine-exposed animals reached thermoneutrality faster, or if our untreated animals did so more slowly than is typically reported (Blumberg et al., 1997).

The pattern of thermoregulatory effects shown here would suggest non-metabolic mechanisms, and potentially point to cardiac or circulatory system effects. Although BAT is located throughout the body, the large interscapular depot of BAT, which is responsible for the delivery of warm blood to the heart and the consequent modulation of cardiac rate (Blumberg et al., 1997; Sokoloff et al., 1998), is of particular importance, as the heart acts as a pumping mechanism to distribute the heat generated by BAT throughout the body via the circulatory system. Our lack of group differences in *T*_IS−Back_ (our measure of BAT thermogenesis) fit with the lack of effect on cardiac rate or R-R Intervals shown here. The startling similarity of the curves shown in Figure 9 attests to this. Instead, cardiac stroke volume, blood pressure, or other circulatory system characteristics may be more significant contributors, but were not measured here. The impact of cocaine on cardiac mass reported here, although minor, implies that stroke volume may be one of a multitude of factors contributing to the thermoregulatory effects of cocaine.

While the mechanism of the alteration in body temperature differences found on PND 3 and 5 may be unclear, this difference likely has implications for the development of other physiological systems and could be detectable by the affected pup's mother, influencing her behavior. A feedback system exists between the pup and mother, such that maternal heat is transferred to pups during close contact, but pups also act as a source of heat for mothers (Woodside and Jans, 1988). Thus, not only does a mother have incentive to isolate hyperthermic pups to reduce the body temperature of the pups, but also to reduce her own body temperature. Abnormally warm pups may offset this feedback loop in favor of reduced maternal attention and increased isolation. Indeed, cocaine-exposed pups have been shown to receive less direct contact from dams, regardless of dam drug exposure (Johns et al., 2005). In theory, the inverse relationship between these two variables (heat and maternal attention) seems in line with the thermal data reported here on PND 3, and may present a potential factor in the patterns of maternal care deficits reported earlier (Johns et al., 2005), although this was not directly tested in the present study (but should be in future work).

The strongest relationship found in our data was that the thermal state of a pup also contributes to the production of ultrasonic vocalizations in the early postnatal period; perhaps especially those vocalizations elicited by thermal mechanisms (eg laryngeal braking). The associations found here between both the weight of the pup and the temperature of the pup and its likelihood of vocalizing suggest that these calls were at least in part produced via such mechanisms. The data found here also indicate that the “body temperature” of the pup (as measured by *T*_Back_) also influences the sonic characteristics of the cry produced, and that this relationship changes with the age of the pup, likely reflecting a dynamic relationship between the size of the respiratory passages and the contractile force of the diaphragm.

Aside from physiological mechanisms (i.e., thermoregulation), pups may also be experiencing psychological stress related to the thermal challenge itself or resulting from isolation from littermates and their dam. In addition to the number of vocalizations produced, a number of other attributes were found to be altered by the thermal state of the pup in all treatment groups, such as the frequency, amplitude, and the variation in frequency of the call. While it is possible that such elements are the result of changes in the physiological mechanisms of vocalization production, it is also likely that such elements reflect the psychological state of the pup. Considering the ages studied here are within the “stress hypo-responsive period” (Lupien et al., 2009), such differences in mechanism might be reflected in the age-related differences between these factors in cocaine-exposed and control animals. In older animals with intact stress systems, applying psychological stress often results in vocalization production (Sánchez, 2003); however, cocaine-exposed animals may develop the necessary biological systems at different rates, leading to differences in vocalizing. Additionally, considering the stress of isolation alone can result in changed vocalization patterns (Kraebel et al., 2002; Shair et al., 2003), it is possible that the stress of isolation differs between treatment groups, which would explain our differences in vocalizations without apparent differences in thermoregulation.

In the context of the broader literature, some common themes have emerged in the study of early pup USV production, demonstrating primarily a decreased number of USVs following prenatal drug insult (Winslow and Insel, 1990; Kehoe and Shoemaker, 1991; Hahn et al., 2000; Tattoli et al., 2001; Antonelli et al., 2005) or malnutrition (Tonkiss et al., 2003). However, these studies used poorly controlled thermal environments and focused simply on the number of vocalizations emitted, thus limiting their comparability to the results presented here. Prenatal cocaine exposure in mice has been shown to increase the starting pitch of calls following a mild thermal challenge, but these effects were dependent upon the genotype of the subject (Hahn et al., 2000) and may not generalize to rats. Regardless, the results presented here and elsewhere (Cox et al., 2012) demonstrate a consistent pattern of altered USV production following prenatal cocaine, with effects dependent upon the age of the animal and the stimulus used to elicit vocalizations.

The data reported here must be interpreted with caution. Considering the deficits in maternal attention reported for cocaine-treated pups in the early postpartum period, without using a cross-fostered group it is difficult to separate the respective contributions of prenatal cocaine and maternal cocaine treatment. Indeed, it is likely that altered maternal care by cocaine-treated and perhaps saline-treated dams contributes to the dynamic differences in pup vocalization and thermoregulation. In order to address these issues, cross-fostering studies are needed. The PND 1 vocalization data presented here resonate with earlier cross-fostering studies showing pups with cocaine exposure elicit poor care from even untreated mothers during the very early postnatal period (Johns et al., 2005); however, the relatively low magnitude of differences we report here suggests that there are more factors involved than shown here. Other pup characteristics (such as odor) clearly play a role in determining care (Okabe et al., 2013) and need to be examined. It is probable that thermal state can also influence other pup behaviors, interacting with other cues to effectively elicit care. Additionally, it will be important to determine differences in stress-related effects aside from those attributable to cocaine treatment alone, and how these effects interact to further alter development.

## Author contributions

Matthew S. McMurray provided funding, designed experiments, collected and analyzed data, and prepared the manuscript. Philip S. Zeskind assisted in experimental design, analyzed data, and provided input on manuscript. Stephanie M. Meiners assisted in experimental design, collected and analyzed data, and provided input on manuscript. Kristin A. Garber assisted in experimental design and analyzed data. Hsiao Tien analyzed data and provided input on the manuscript. Josephine M. Johns provided funding and assisted in experimental design, data analysis, and manuscript preparation.

## Conflict of interest statement

The authors declare that the research was conducted in the absence of any commercial or financial relationships that could be construed as a potential conflict of interest.
